# The Influence of Medial Comminution in the Treatment Choice of Radial Head Fracture

**DOI:** 10.5704/MOJ.2011.019

**Published:** 2020-11

**Authors:** G Touloupakis, E Biancardi, E Theodorakis, S Ghirardelli, F Ferrara, F Gherlinzoni, G Antonini

**Affiliations:** 1 Department of Orthopaedics and Traumatology, San Carlo Borromeo Hospital, Milan, Italy; 2 Department of Orthopaedics and Traumatology, AAS 2 Bassa Friulana-Isontina, Gorizia, Italy; 3 Department of Orthopaedics and Traumatology, Fatebenefratelli Hospital, Milan, Italy

**Keywords:** radial head fracture, radial head replacement, elbow joint, comminuted fractures, fracture fixation

## Abstract

**Introduction:**

The aim of our retrospective study was to investigate the role of the medial side involvement in the treatment choice of radial head fractures.

**Materials and Methods:**

We searched the databases of our institutions for the surgical procedures diagnosed as "fracture of the radial head" and for the procedures related to "prosthesis of the radial head" and "osteosynthesis of the radial head" in the period from May 2014 to October 2017. The fractures were first classified according to the Mason classification . We then allocated the patients into three study groups according to the site of the fracture, either the medial or lateral side of the radial head : Group A, with an isolated lateral fracture of the radius head; Group B1, with a medial fracture of the radius head with two medial fragments; and Group B2, with a medial fracture of the radius head with multiple medial fragments. We performed a multivariate analysis to identify statistically significant correlation between the pre-operative classifications of Mason and our study, the type of surgical procedure, and the clinical outcome.

**Results:**

Mayo Elbow Performance (MEP) scores determined at the final follow-up of the study (mean 16.6 months, range 12-26 months) was excellent in 17 patients (4 in Group A, 6 in Group B1 and 7 in Group B2), and good in 12 patients (3 in Group A, 7 in Group B1, and 2 in Group B2). One patient showed a poor result in MEP score probably because of an infection and implant removal.

**Conclusion:**

Regarding medial fractures of the radial head, our study showed satisfactory results with a radial head prosthesis for comminuted or multifragmentary radial head fractures. For surgeons with advanced elbow fracture expertise, osteosynthesis could be attempted in a fracture pattern that involved only two medial fragments.

## Introduction

The Mason classification was initially published in 1954 and subsequently modified by Hotchkiss and Broberg-Morrey^1^. Its primary purpose was to guide surgical treatment according to the injury pattern of the radial head fractures. However, there was no clear correlation between a surgical intervention proposed and the actual subtype of the fracture. Treatment decisions could therefore be guided by, but not be rigidly based on, the classification.

**Fig. 1:**
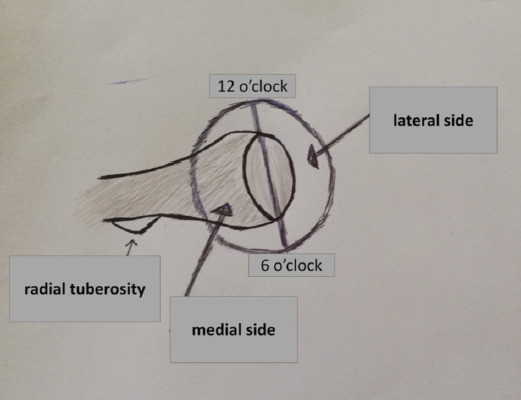
The radial tuberosity (6 o’clock) was used as a reference point to distinguish fragments as lateral and medial ones (right elbow).

The crucial role of the medial involvement in the fracture of the radial head lacked careful consideration in the Mason classification. This would be its principal limitation as a surgical guide. The aim of our study was to investigate the role of the medial side involvement in the treatment choices of radial head fractures (Fig.1).

## Materials and Methods

This is a retrospective study. We searched the databases of our institutions for the surgical procedures diagnosed as "fracture of the radial head" and for the procedures "prosthesis of the radial head" or "osteosynthesis of the radial head” from May 2014 to October 2017.

The subjects in the study were patients who had a previous radial fracture, Type II, III or IV according to the Mason classification, and routinely diagnosed by two standard radiograph projections of the elbow, with anteroposterior and lateral views, and a 3-D CT scan. The exclusion criteria were the following: (1) Comorbidity Severity Score with ASA Physical Status Classification (ASA) ≥4; and (2) Postoperative follow-up shorter than twelve months.

The radiograph images of the elbow of all the eligible patients were obtained from the picture archiving and communication system (PACS) in our institutions with at least two standard projections, anteroposterior and lateral views of the elbow. In addition, CT scans of the elbow were available to evaluate the size and characteristics of the radial head fracture, and the involvement of the articular surface.

In cases with elbow dislocation, post-reduction splinting CT scans were obtained. All radiographic images were supervised by two surgeons, a chief orthopaedic resident and a consultant orthopaedic surgeon with extensive experience in upper limb fractures.

All CT scans with 1.0mm slices were assessed to define the position and size of the fracture fragments. We quantified the extent of the fracture through the axial cut that best showed the largest diameter of the radial head. Similarly, the axial cut that best showed the maximum extension of the radial tuberosity was used as an angular reference for the localisation of the fractures. We considered it appropriate and very practical to define the apex of the radial tuberosity as the "6 o'clock" reference point , as an easily identifiable marker, in pre-operative, intra-operative, and post-operative CTs, and was clear of any involvement in the fractures in our case series. Through software, we superimposed on the selected slices a graduated clock-like circumference to define the location of the fractures. We defined a fracture as medial if placed medially to the straight line that connected the 6 to 12 o'clock position. In the same way, we defined a fracture as lateral if it was lateral to the straight line connecting the 6 to 12 o'clock position^2^. Measurements were performed using the angle measuring tool provided by PACS.

All the radial head fractures were classified according to Mason's classification. We analysed the characteristics of the fragmentation and identified those fractures with medial involvement of the articular surface of the radial head, as described previously. When a medial fracture was detected pre-operatively we defined the number of the medial fragments by looking at the fracture line: one fracture line corresponded to two fragments; two or more fracture lines corresponded to three or more fragments.

We then proceeded to classify the patients according to the side of the radial head involved in the fracture. There were three groups: Group A, with isolated lateral fracture of the radius head; Group B1, with medial fracture of the radius head with two fragments; and Group B2, with medial fracture of the radius head with multiple fragments.

For each procedure, all types of implants were always available to the surgeon: anatomical plates, headless screws, cannulated screws, threaded wires, and radial head prostheses. The selection of the surgical option was made only after an intra-operative verification of the capsule-ligament stability, the bone quality, the size of the fragments and the expected stability of the synthesis to be performed.

We performed a multivariate analysis to identify a statistically significant correlation between the pre-operative classifications (Mason and the Medial / Lateral proposal), the type of surgical procedure, and the clinical outcome using the statistical package for Microsoft Excel (Microsoft Corporation) and the open-source statistical software R (the R Foundation for Statistical Computing). In our analysis, we compared clinical outcome according to the MEP score, surgical effectiveness between fracture types and/or treatment choices.

As data were provided from trauma patients, it was not possible to compare the MEP score with the pre-operative status. Therefore, we focused on post-operative data at six months and at one year, to find the significant differences in the study population.

The median age of the patients was calculated and reported. All patients were followed-up after surgery as outpatients with post-operative radiograph examinations according to our protocol, with anteroposterior and lateral views of the radial head at five weeks, three months, six months, and one year, and after that at regular intervals, depending on the overall clinical status and clinical requirements. At each follow-up, a clinical examination was conducted and a record made of the bone union and the presence of any of the post-operative complications of pseudoarthrosis, loosening of the radial head prosthesis, infections and nerve lesions.

The functional outcome was quantified using the Mayo Elbow Performance (MEP) Index. All patients were prescribed with indomethacin 25mg thrice daily, and omeprazole 20mg daily for three weeks, as heterotopic ossification prophylaxis. Physiotherapy was recommended to patients on discharge for the necessary period.

## Results

The cohort of patients that met our inclusion criteria was composed of 30 subjects with 12 males and 18 females. The median age at the time of surgery was 50.5 years old, (range 26 to 70 years). Fourteen patients had a fracture of the right arm, and sixteen had the fracture in the left arm.

Group A consisted of seven patients: four of them were Mason type II, two patients were Mason type III, and one patient was Mason type IV. All the patients in this group underwent an osteosynthesis of the radial head. Different types of osteosynthesis were performed, with one to three screws; in four cases comprising of three Mason type II and one Mason type III. Locking compression plating was performed in three cases: , one Mason type II; one Mason type III; and one Mason type IV.

Group B consisted of 23 patients, and all received a prosthesis. Thirteen patients were included in Group B1: two were Mason type II, six were Mason type III, and five were Mason type IV. Five patients received a prosthesis, in two Mason type IV and three Mason type III patients. Eight patients underwent an osteosynthesis. One to three screws were used in six patients: two were Mason type II, one was Mason type III, and three were Mason type IV. Locking compression plates were used in two patients with Mason type III fractures. Ten of these patients were placed in Group B2; seven of them were Mason type III, and three were Mason type IV.

Fifteen patients who presented with medial fragmentation of the radius head underwent a radial head replacement: ten were Mason type III, and five were Mason type IV. There were no intra-operative complications.

Post-operatively, one patient acquired an infection of the surgical site, and the prosthesis was removed three months after the initial surgery. Periprosthetic bone lysis was observed in four cases, twelve months after the surgery. In all the cases treated with osteosynthesis, bone healing was documented at the twelve-month follow-up. No cases of pseudarthrosis or nerve lesions were seen. MEP scores determined at the final follow-up of the study, (mean 16.6 months, range 12-26 months) was excellent in seventeen patients, with four in Group A, six in Group B1 and seven in Group B2; and was good in 12 patients, with three in Group A, seven in Group B1, and two in Group B2. One patient showed a poor MEP score probably because of acquired infection and implant removal.

Data were checked for normality through the Kolmogorov-Smirnov test and they showed a normal distribution, except in the MEP score at six months in the prosthesis group, for which the test demonstrated a likely not normal distribution (skewness: 1.85m,; kurtosis: 3.68, p=.03645), requiring additional non-parametrical tests.

Mean overall MEP score at six months was 85.76 ±8.7, while at one year, it was 88.6 ±8.48, with a mean improvement of 2.83±2.5 points. T-test was performed, and the difference was statistically significant (p<0.001).

Data were then grouped to show any difference between the three treatment groups: prosthesis, screw(s) synthesis, plate synthesis; and the three diagnosis groups, A, B1, B2. In the treatment groups, the prosthesis showed an MEP score at six months of 85.06 ± 10.59 and at one year, a score of 87.93 ±10.55; the screw(s) at six months was 86.7 ±7.07 and at one year 89.3 ± 6.3; while the plate osteosynthesis was 86 ± 7.07 at six months and 89.2 ±6.2 at one year. The mean improvement from six to twelve months was thus 2.8 ±3.15 for the prosthesis (p=0.003, Wilcoxon test); 2.6 ±1.34 for the screw(s) (p<0.001, T-test) and 3.2 ±3.03 for the plate (p=0.038, T-test). The difference was, therefore, statistically significant in all these groups.

Kruskal Wallis test found no significant difference in the outcomes between the patient groups. Group A showed an MEP score at six months of 87 ± 7.37 and one year of 89.85 ± 6.64; Group B1 at six months was 85.77 ±6.61 and at one year 88.3 ±6.87; while Group B2 was 85.9 ±12.14 at six months and 88.1 ±11.75 at one year. Mean improvement was 2.85 ± 1.86 for Group A (p=0.003, T-test), 2.53 ±1.89 for B1 (p<0.001, T-test) and 3.2 ±3.76 for B2 (p=0.01, T-test). The difference was therefore statistically significant in the patient groups as well as for the treatment groups. However, the final determination with an analysis of variance (ANOVA) found no significant difference in outcomes in patients grouped by fracture type.

## Discussion

Mark Mason, in his 1954 article, reported that the elbow joint tolerated trauma quite badly; even a minor injury could lead to a loss of the range of joint movement. In his conclusion, he stated “If in doubt, resect” as the axiom in the treatment of fractures of the head of the radius^3^.Today, knowledge of the elbow biomechanics and the development of dedicated implants have changed the treatment approaches, and resection is no longer the common option for surgical treatment^4-5^. The classification of fractures he proposed is still very popular. Broberg-Morrey and Hotchkiss proposed several modifications, but Mason’s name remains synonymous with the classification. One of the limitations of the modified Mason’s classifications, is the lack of consideration given to the medial comminution and medial extensions of the fragmentation of the head of the radius. The planning of surgical treatment, should not be based only on the Mason classification as there is a body of literature supporting different types of surgical procedures. Furthermore, the classification of a fracture based on the number of major fragments can have different results depending on whether a 2D or 3D CT scans is used^6^. Moreover, there is no consensus on the outcome of a fixation of a standard Mason type-III, by using either K-wires or mini-screws, versus a radial head prosthesis.

Several studies published over the last fifteen years suggest that an open reduction and internal fixation of the radial head is effective for partial articular fractures when major fragments are less than three. Comminuted fractures may be better treated by radial head prosthetic replacement or by radial head excision^7-11^.

To our knowledge, our study is the first to investigate the role of the anatomical position of the fragment in the choice of surgical treatment. All patients with an exclusively lateral involvement of the radial head, regardless of the number of fragments, were treated with open reduction and internal fixation (ORIF). The results were excellent at six months, with further improvements at 12 months. Based on our experience, we suggest treating with ORIF for any of these cases, even with an on-the-table technique. Cooney considered a prosthetic solution as a valid option for more unstable, comminuted displaced radial head fractures that could not be reconstructed^12^. Replacement also is indicated in patients with comminuted radial head fractures that have associated lateral and medial collateral ligaments lesions^13^. Ring underlined the importance of the restoration of elbow and forearm stability when unstable displaced fractures of the radial head occurred in association with other fractures or ligament injuries. In such cases, replacement of the radial head with a metal prosthesis might be preferable^4^.

In our study, the only valid treatment for medial fractures with more than two fragments is the replacement with a radial head prosthesis. If there are only two medial fragments, it is possible to proceed with an osteosynthesis, keeping in mind that the operating times are considerably longer and the technical skill of a specialist in elbow surgery is required.

The surgical approach is crucial. A mini Kocher approach, which is the most popular among non-elbow-specialised surgeons, does not offer an adequate visualisation of the medial side of the radial head. Other approaches fit better this need to provide a better view, like the extended Kocher, the extensor digitorum communis split, and the Kaplan technique, but these require more expertise of the operator. All the radial head fractures with purely lateral fragmentation can be treated by reduction and fixation, without the necessity to proceed with a replacement. We, therefore, recommend an attempt to fix the fractures, as this is always preferable, even with an “on- the- table” technique, when only lateral fragmentation is present, as a prosthesis is typically associated with reabsorption and technical difficulties.

We experienced technical pitfalls when a fracture was medial, and combined with multi-fragmentation. Nevertheless, all our prosthetic implants have given a satisfactory result except in the one case complicated by infection and an unavoidable low functional MEP score. We, therefore, conclude that implants should be preferred in fragmented medial fractures, even when a slight bone loss around the stem is visible in radiograph images.

## Conclusion

In conclusion, we would like to underline the influence of the medial fragmentation as one of the most important factors to consider when dealing with isolated radial head fractures. We recommend the treatment of isolated lateral radial head fractures by fixation. Regarding medial fractures of the radial head, our study showed satisfactory results with radial head prosthesis for comminuted or multifragmentary fractures. However, for surgeons with advanced elbow fracture expertise, osteosynthesis could be attempted in a fracture pattern that involved only two medial fragments. The establishment of a multi-centre study is needed to evaluate and confirm the results of our retrospective study, which is based on a small sample.
